# TCR Gene Therapy: Challenges, Opportunities, and Future Directions

**DOI:** 10.3390/cells9122567

**Published:** 2020-12-01

**Authors:** Hans J. Stauss, Maxine G. B. Tran

**Affiliations:** 1 Division of Infection and Immunity, Institute of Immunity and Transplantation, University College London, Royal Free Hospital, London NW3 2PF, UK; 2 Division of Surgery and Interventional Science, University College London, Royal Free Hospital, London NW3 2PF, UK; m.tran@ucl.ac.uk

Adoptive immunotherapy with gene-engineered T cells has provided new treatment options for cancer patients. The most successful strategies have involved the engineering of T cells expressing chimeric antigen receptors (CARs) directed against differentiation antigens expressed in haematological malignancies. To date, clinical trials with TCR gene-engineered T cells have not yet demonstrated the clinical efficacy seen with CAR-T cells directed against CD19-positive blood cancers. However, this Special Issue demonstrates some of the advantages of TCR engineering of T cells to target cancer antigens, including mutated neoantigens, that cannot be reached by the CAR technology. In this issue, the authors provide insights into the nature of cancer proteins that can be targeted by TCR engineering, demonstrate that virus-driven human cancers can be targeted with TCR gene therapy, explore the role of CD4+ helper T cells and CD8+ cytotoxic T cells, show how TCRs from CD8+ T cells can be functional in CD4+ T cells, and finally discuss how targeting of the tumour microenvironment may enhance the efficacy of TCR gene therapy of solid cancer. This Special Issue provides detailed insight into the opportunities of TCR gene therapy and how it may enable the development of highly specific immunotherapies for the treatment of solid cancer.

## 1. The Case for TCR Engineering

The paper by Crowther and colleagues provides an excellent comparison of the CAR and TCR technology platforms that are used to redirect the specificity of patient T cells against selected target proteins present in cancer cells [1]. Over recent years, the CAR technology has evolved to improve T-cell activation by adding a combination of signalling domains into the intracellular portion of the CAR constructs. In addition, the CAR engineering has been combined with the expression of effector cytokines to produce “T-cells Redirected for Universal Cytokine Killing”, called TRUCKs. However, these technologies have not overcome the major limitations of the CAR platform, which is the inability to target proteins that are expressed inside cancer cells. Considering that 80% of all cellular proteins are expressed intracellularly, only 20% of potential cancer proteins can be reached by the CAR technology. Interestingly, a number of groups have shown that this limitation can be circumvented by generating CARs against peptides presented by HLA molecules, thus mimicking the natural specificity of TCRs [2].

Gaissmaier and colleagues provide an excellent overview of the challenges and bottlenecks of TCR gene therapy [3]. A major benefit of TCRs is that they can initiate T-cell activation when target cells present as few as 1–100 peptide epitopes. This indicates that the sensitivity of TCR-redirected T cells is much greater than that of CAR-redirected T cells, which require at least 1000 epitopes in target cells to trigger activation. The high level of sensitivity enables TCR targeting of cancer neoantigens, which arise from somatic mutations producing mutant proteins that are only present in tumour cells and not in normal tissues. The density of HLA molecules presenting mutated epitopes is usually less than 100 copies per cell, which is below the epitope density required for CAR activation. Gaissmaier et al. discuss the challenges of identifying TCRs that are specific for cancer neoantigens and provide a comprehensive overview of how cutting-edge technologies and bioinformatic strategies can be exploited to achieve neoantigen-specific TCR gene therapy.

There is currently much effort to generate “off the shelf” cell products that could be used in multiple patients. Moving from autologous T-cell products that are unique for each individual patient to a generic T-cell product that can be utilised in many patients would be a major step towards providing engineered T-cell therapy at a large scale and mitigating current prohibitive costs. Mornadi et al. discuss the advantages of engineering γ/δ T cells and NKT cells for therapy in the allogeneic setting [4]. The major advantage of both cell types is that they do not express a polyclonal endogenous α/β TCR repertoire which avoids mispairing with an introduced α/β TCR, and the lack of an endogenous repertoire also eliminates allo-reactivity and the risk of graft versus host disease after cell transfer into allogenic hosts. In addition, engineered γ/δ T cells and NKT cells may benefit from the cancer-specificity of the introduced α/β TCR, and also from the anti-cancer activity of endogenous γ/δ and invariant NKT TCRs. However, although γ/δ T cells, NKT and NK cells are unlikely to mediate alloreactive GvHD, additional engineering will be required to remove HLA molecules and other immunogenic antigens that would stimulate an immune response after adoptive transfer into patients, which would result in the elimination of the transferred cells.

## 2. Engineering CD4 and CD8 T Cells with the Same TCR

Three papers in the Special Issue have explored the use of TCR-redirected CD4+ T cells for adoptive T-cell therapy. Klobuch et al. have isolated TCRs specific for the human HLA-DPB1 antigen [5]. This MHC class II antigen is a potential target for anti-leukaemia immunity in patients undergoing allogeneic haematopoietic stem cell transplantation from unrelated donors. The authors demonstrate that human CD4+ T cells redirected with an HLA-DPB1-specific TCR show anti-leukaemic activity in vitro and also in a xenogeneic in vivo model, while CD8+ T cells expressing the same TCR show reduced anti-leukaemic activity. However, the authors found that optimising TCR function in CD4+ T cells is associated with the recognition of HLA-DPB1-positive fibroblast, raising the concern that these T cells might cause GvHD in patients. The study by Schober et al. explored a different strategy to produce cancer-specific CD4+ T cells [6]. This group isolated HLA class-I-restricted TCRs specific for six-transmembrane epithelial antigen of the prostate 1 (STEAP1), an antigen that is expressed not only in prostate cancer but also in Ewing’s sarcoma. The authors found that TCR-redirected CD4+ T cells and CD8+ T cells both controlled local tumour growth in a xenogeneic Ewing’s sarcoma model, but only the CD8+ T cells were able to reduce the development of metastatic lesions. Both studies demonstrate that TCRs isolated from CD4+ T cells or from CD8+ T cells are only fully functional when they are expressed in the T-cell subset from which they were derived. This is discussed in detail by Sillito et al., who provide an overview of the role of the CD4/CD8 coreceptors in enabling optimal TCR recognition of peptides presented by MHC class I and class II molecules [7]. The co-transfer of class-I-restricted TCR plus CD8 coreceptor and class-II-restricted TCR plus CD4 coreceptor provides a strategy to achieve optimal TCR function in all T cells. An alternative strategy is to enhance TCR affinity, which can compensate for the reduction in avidity when the relevant CD4 or CD8 coreceptor is missing. 

The importance of TCR affinity and T-cell avidity is addressed in the paper by Campillo-Davo et al. [8]. Affinity improvements of TCR for the cognate peptide/MHC complex can be achieved by the introduction of mutations into the CDR regions of the TCR. The natural affinity of TCRs is approximately 1000-fold lower than the affinity of antibodies binding to their target antigen. Artificial affinity maturation can be used to produce TCRs that have antibody-like affinities. Surprisingly, these artificial TCRs are no longer able to trigger T-cell activation when the antigen density of MHC presented peptides is low [9]. Affinity maturation also changes the fine specificity of TCRs, which may result in unexpected cross-reactivity and in vivo toxicity. Hence, current artificial affinity maturation strategies remain problematic and require refinement to improve the functional efficacy of TCRs.

## 3. Technologies to Improve TCR Gene Therapy

To date, TCR transgenic T cells most often express the introduced TCR in addition to the endogenous TCR. The expression of two TCRs in the same T cells has two major disadvantages; mispairing between introduced and endogenous TCR chains can produce unknown specificities and toxicity, as evidenced in mouse models when T cells are adoptively transferred in vivo [10]. Secondly, the endogenous and introduced TCRs compete for access to CD3 molecules that are essential for TCR expression on the cell surface; this competition results in reduced TCR expression. Schober et al. present an excellent insight into cutting-edge CRISPR-Cas9 engineering technology used to insert the introduced TCR into the endogenous TCR gene locus [11]. This results in the disruption of the endogenous TCR and the physiological expression of the introduced TCR from the endogenous TCR promoter. Schober and colleagues discuss the advantages of the “physiological” expression of introduced TCR, while also pointing to the remaining challenges of this TCR replacement technology.

The paper by Rath et al. provides a comprehensive overview of the clinical trial experience with TCR-engineered T cells, while also reviewing how synthetic biology approaches may further enhance the efficacy of TCR gene therapy [12]. Synthetic biology can be employed to convert inhibitory receptors into a stimulating molecule by exchanging intracellular signalling domains, and CRISPR technology presents a powerful opportunity to enable the disruption of genes encoding proteins that negatively impact tumour protective effector function and memory formation of T cells.

## 4. TCR Gene Therapy of Virus-Driven Cancer

Two papers illustrate how TCR gene therapy can be utilised as a treatment option for malignancies associated with infection by Epstein–Barr Virus (EBV) and hepatitis virus. Healy et al. provide a summary of currently known peptide epitopes in HBV, HCV, and HEV that can be targeted by TCRs [13]. The authors discuss the rationale for TCR gene therapy of hepatocellular carcinoma by selecting viral epitopes that are expressed in cancer cells. The major challenge of this approach is to achieve control viral infection of healthy liver tissue without causing clinical symptoms of liver damage. 

The contribution by Munz covers the therapeutic options to treat EBV infection and EBV-driven cancer [14]. A TCR gene therapy approach to target latent EBV proteins expressed in transformed cells, and also early EBV proteins associated with active EBV replication, may result in the elimination of cancer cells while also controlling latent EBV infection. Munz provides a summary of ongoing clinical trials using T cells engineered with TCRs or CARs to treat EBV-associated cancer. While all TCR gene therapy trials target EBV proteins, most CAR trials target normal cellular proteins, such as CD19 and CD30, which are expressed in transformed B cells.

This Special Issue provides a comprehensive cutting-edge overview of current and future immunotherapy of cancer with TCR-engineered patient T cells. The TCR gene therapy approach is not in competition with CAR–T-cell therapy, but instead, it extends the scope of T-cell engineering to include intracellular proteins and mutated cancer neoantigens that are difficult to target with the conventional CAR technology platform.

## References

[1] Crowther M.D., Svane I.M., Met O. (2020). T-Cell Gene Therapy in Cancer Immunotherapy: Why It Is No Longer Just CARs on The Road. Cells.

[2] Oren R., Hod-Marco M., Haus-Cohen M., Thomas S., Blat D., Duvshani N., Denkberg G., Elbaz Y., Benchetrit F., Eshhar Z. (2014). Functional comparison of engineered T cells carrying a native TCR versus TCR-like antibody-based chimeric antigen receptors indicates affinity/avidity thresholds. J. Immunol..

[3] Gaissmaier L., Elshiaty M., Christopoulos P. (2020). Breaking Bottlenecks for the TCR Therapy of Cancer. Cells.

[4] Morandi F., Yazdanifar M., Cocco C., Bertaina A., Airoldi I. (2020). Engineering the Bridge between Innate and Adaptive Immunity for Cancer Immunotherapy: Focus on gammadelta T and NK Cells. Cells.

[5] Klobuch S., Hammon K., Vatter-Leising S., Neidlinger E., Zwerger M., Wandel A., Neuber L.M., Heilmeier B., Fichtner R., Mirbeth C. (2020). HLA-DPB1 Reactive T Cell Receptors for Adoptive Immunotherapy in Allogeneic Stem Cell Transplantation. Cells.

[6] Schober S.J., Thiede M., Gassmann H., Prexler C., Xue B., Schirmer D., Wohlleber D., Stein S., Grunewald T.G.P., Busch D.H. (2020). MHC Class I-Restricted TCR-Transgenic CD4(+) T Cells Against STEAP1 Mediate Local Tumor Control of Ewing Sarcoma In Vivo. Cells.

[7] Sillito F., Holler A., Stauss H.J. (2020). Engineering CD4+ T Cells to Enhance Cancer Immunity. Cells.

[8] Campillo-Davo D., Flumens D., Lion E. (2020). The Quest for the Best: How TCR Affinity, Avidity, and Functional Avidity Affect TCR-Engineered T-Cell Antitumor Responses. Cells.

[9] Thomas S., Xue S.A., Bangham C.R., Jakobsen B.K., Morris E.C., Stauss H.J. (2011). Human T cells expressing affinity-matured TCR display accelerated responses but fail to recognize low density of MHC-peptide antigen. Blood.

[10] Bendle G.M., Linnemann C., Hooijkaas A.I., Bies L., de Witte M.A., Jorritsma A., Kaiser A.D., Pouw N., Debets R., Kieback E. (2010). Lethal graft-versus-host disease in mouse models of T cell receptor gene therapy. Nat. Med..

[11] Schober K., Muller T.R., Busch D.H. (2020). Orthotopic T-Cell Receptor Replacement—An “Enabler” for TCR-Based Therapies. Cells.

[12] Rath J.A., Arber C. (2020). Engineering Strategies to Enhance TCR-Based Adoptive T Cell Therapy. Cells.

[13] Healy K., Pasetto A., Sobkowiak M.J., Soon C.F., Cornberg M., Aleman S., Sallberg Chen M. (2020). Chronic Viral Liver Diseases: Approaching the Liver Using T Cell Receptor-Mediated Gene Technologies. Cells.

[14] Munz C. (2020). Redirecting T Cells against Epstein-Barr Virus Infection and Associated Oncogenesis. Cells.

